# Systemic Treatments for Noninfectious Vitreous Inflammation

**DOI:** 10.1155/2013/515312

**Published:** 2013-11-20

**Authors:** Angela Jiang, Jillian Wang, Malav Joshi, John Byron Christoforidis

**Affiliations:** ^1^Department of Ophthalmology, The Ohio State University Wexner Medical Center, 915 Olentangy River Road, Suite 5000, Columbus, OH 43212, USA; ^2^Department of Ophthalmology, The University of Arizona Medical Center, 655 North Alvernon Way, Suite 108, Tucson, AZ 85711, USA

## Abstract

Vitreous inflammation, or vitritis, may result from many causes, including both infectious and noninfectious, including rheumatologic and autoimmune processes. Vitritis is commonly vision threatening and has serious sequelae. Treatment is frequently challenging, but, today, there are multiple methods of systemic treatment for vitritis. These categories include corticosteroids, antimetabolites, alkylating agents, T-cell inhibitors/calcineurin inhibitors, and biologic agents. These treatment categories were reviewed last year, but, even over the course of just a year, many therapies have made progress, as we have learned more about their indications and efficacy. We discuss here discoveries made over the past year on both existing and new drugs, as well as reviewing mechanisms of action, clinical dosages, specific conditions that are treated, adverse effects, and usual course of treatment for each class of therapy.

## 1. Introduction

Vitreous inflammation, or vitritis, may result from many causes, including both infectious and noninfectious. Epidemiologic studies indicate that uveitis accounts for 2–10% of prevalent blindness in the European and North American population and is therefore an underrated and significant public health problem [1]. Infectious etiologies include bacterial Lyme, syphilis, or *Bartonella*; viruses HSV, VZV, and CMV, and a variety of fungal and parasitic causes. Noninfectious etiologies include rheumatologic and autoimmune processes, examples being sarcoidosis, systemic lupus erythematosus, multiple sclerosis, and Behcet's disease. However, idiopathic vitritis without associated systemic disease is most common. Vitritis is sometimes visionthreatening, due to sequelae such as cystoid macular edema (CME), vitreous opacities, and retinal detachment, ischemia/neovascularization, or pigment epithelium changes. Glaucoma and cataracts may also form. With such serious sequelae, there are multiple methods of systemic treatment for vitritis. On the other hand, mild vitritis without vasculitis or CME can sometimes be followed closely without any treatment. The goal of all types of treatment is to rapidly alter and stop the course of intraocular inflammation but at the same time minimize any side effects from these systemic drugs. We reviewed these treatment categories last year, but, even over the course of just a year, many therapies have made progress, as we have learned more about their indications and efficacy [2].

## 2. Initial Treatment: Corticosteroids

The first line of treatment for noninfectious uveitis is corticosteroids. This group of drugs is used to suppress inflammation, either systemically or intraocular. The accepted algorithm for treatment begins with topical glucocorticoids, with frequency depending upon severity and not necessarily etiology. However, topical corticosteroids have been shown to have poor penetration into the posterior segment and are thus not used often for posterior segment disease; they are more commonly used to reduce anterior chamber inflammation and have only a minor effect on vitreous inflammation [3]. Oral or intravitreal corticosteroids are therefore used to treat cases of posterior segment disease. Oral prednisone (1 mg/kg/day with gradual tapering) is often the first therapeutic agent used [4]. 

Intravitreal delivery systems include injection or implantation of periocular or intravitreal steroid compounds (triamcinolone acetonide) [5]. There are several different types of systems, either nonbiodegradable or biodegradable; a more extensive review of drug delivery implants is reviewed in our other paper. Although previous studies raised concern for recurrence of inflammation as intravitreal steroid concentration decreases, some recent trials elude that this may no longer be the case [6]. Patients undergoing treatment with local delivery methods will usually have minimal adverse events. It has however been reported that localized side effects may occur, such as cataract formation, increased intraocular pressure, and transient vitreous hemorrhage.

On the other hand, those undergoing systemic corticosteroid therapy often encounter nonocular adverse events, such as arthralgia and hypertension. Other common complications range from those affecting the musculoskeletal system (osteoporosis, aseptic bone necrosis, and myopathy), gastrointestinal system (ulcers and pancreatitis), endocrine (hyperglycemia and cushinoid features), infectious, (delayed wound healing, secondary infection, and reactivation of latent herpes simplex or tuberculosis), or even psychosis. If patients develop adverse effects, or are refractory to treatment with corticosteroid therapy, switching to an intravitreal delivery system or considering systemic immunosuppressive therapy is indicated [7].

## 3. Immunosuppressive Treatment

Systemic immunosuppressive therapy can either supplement or completely replace corticosteroid therapy, for the reasons touched upon above. There are several conditions that have been found to be refractory to corticosteroid treatment but instead respond to immunosuppressives. Examples of these conditions ran the gamut of several autoimmune diseases such as Behcet's, Wegener's, or juvenile idiopathic arthritis-associated uveitis [8]. Other conditions that indicate immunosuppressive therapy are found in Table 1.

There are several categories of immunosuppressive agents: antimetabolites, alkylating agents, T-cell inhibitors/calcineurin inhibitors, and biologic agents. Information about these categories is available in Table 2, while newer biologics and investigations are discussed below.  Table 3 addresses ocular diseases and which groups of immunosuppressive agents are used to treat them.

In general, treatment with immunosuppressives starts after or with corticosteroid therapy, with local treatment attempted before systemic treatment, if the disease process is amenable. Systemic treatment attempts to start with the least toxic medications in the case of mild-moderate disease; methotrexate and cyclosporine are most commonly used after corticosteroids, followed by more antimetabolites. Severe, vision-threatening disease may require the use of biologic or cytotoxic agents, although they are avoided whenever possible due to their severe adverse effects.

### 3.1. Leflunomide

Leflunomide is a noncytotoxic drug that works on both the cellular and humoral immune response. It is most commonly used for systemic rheumatologic diseases, examples being severe rheumatoid or psoriatic arthritis. Ocular use in treating chronic inflammation associated with sarcoidosis is currently under investigation [9]. Recently, Leflunomide was proven as both safe and efficacious for long-term therapy treating chronic anterior uveitis associated with juvenile idiopathic arthritis [10]. Most patients maintained an ocular response to the drug and underwent only a few mild adverse effects. Common adverse effects of Leflunomide include hepatotoxicity with known fatalities, myelosuppression with resulting opportunistic infection and anemia, interstitial lung disease, alopecia, and skin reactions (Stevens Johnson and toxic epidermal necrolysis). Leflunomide is also a teratogen (pregnancy class X), and patients need to be on contraception during treatment. Overall, it is a promising form of treatment, as methotrexate is currently the first and was previously the only choice for patients with juvenile idiopathic arthritis.

### 3.2. Biologic Agents

Biologic agents are one of the newest classes of therapeutic proteins. They were originally developed for preventing organ transplant rejection but were found to be useful for treating systemic inflammatory diseases as well. They are now used off label in treating uveitis, and have been used with some success for refractory cases. Biologic agents' major mechanisms of action all revolve around targeting specific inflammatory molecules, with the goal of inhibiting mediators or cytokines. Examples of these inflammatory mediators include tumor necrosis factor alpha and interleukin-2. Due to their strong immunologic suppression, serious adverse effects revolve around infectious processes or malignancies such as lymphoma. Latent and opportunistic infections are especially important to monitor for and include those such as tuberculosis, histoplasmosis, coccidiomycosis and herpes viruses. 

Biologic agents are categorized into two groups: monoclonal antibodies and fusion proteins. Monoclonal antibodies are further classified and suffixes named based on their regions (either human, murine, or a combination of regions). Fusion proteins are created by joined genes, and are a combination of a receptor and another protein fragment.

#### 3.2.1. Adalimumab

Adalimumab is a recombinant, full-length humanized immunoglobulin directed against tumor necrosis factor (TNF). It is able to bind with both high affinity and specificity to soluble TNF*α* or *β*, thus neutralizing the biological function of TNF, as well as modulating biological responses that TNF is responsible for inducing or regulating [11]. It is currently used with increasing frequency for treating several autoimmune diseases such as Behcet's, juvenile idiopathic arthritis-associated uveitis, Vogt-Koyanagi-Harada (VKH) disease, and birdshot retinochoroidopathy [12–16]. A recent multicenter trial found it to be a useful treatment for patients with refractory uveitis, with a 10-week success rate of 68% [17].

A more recent retrospective analysis of 60 patients, the largest case series to date, showed a positive effect of adalimumab in 82% of these patients with different uveitis types, independent of additional systemic disease [11]. This study found that those who had been treated with infliximab and etanercept with insufficient response were effectively treated with adalimumab in 92% of cases. Another interesting finding was that patients pretreated with other TNF agents still had good results; thus, it is reasonable to switch to another TNF agent if the first was ineffective. In this study, no major infections nor serious complications known to TNF inhibitors (demyelinating disease, reactivation of TB) occurred. This is a significant finding, as adalimumab may thus be a better option than infliximab, although follow-up was short and the study's power would need to be increased in a further study.

Another prospective study evaluated the efficacy and outcomes of using adalimumab to treat uveitis associated with juvenile idiopathic arthritis [18]. Ocular symptom improvement was seen in 76% of cases, with anterior uveitis flare rate reduced after starting treatment. This study also confirmed a lack of serious sideeffects and infections and fewer hypersensitivity reactions than infliximab. Overall, this study concluded that adalimumab was a reasonable adjuvant therapy for treating uveitis.

#### 3.2.2. Rituximab

Rituximab is an antibody that binds CD20, with many effects. Most commonly used in hematologic and autoimmune disorders, it has been found to be effective as a sole treatment for Wegener's uveitis and retinal vasculitis [19, 20]. The value of rituximab in Behcet's disease is yet to be determined, due to limited evidence [21]. In addition, it has also been used with intravenous IgG to treat ocular cicatricial pemphigoid [22].

#### 3.2.3. Tocilizumab

Tocilizumab is a humanized antibody that binds both to IL-6 receptors, originally used for treating rheumatoid arthritis and systemic juvenile idiopathic arthritis [23]. IL-6 has a role in proliferation and differentiation of T- and B-cells, with persistent production demonstrated in chronic inflammatory diseases. Although ophthalmologic usage is currently limited, patients with active posterior uveitis have been found to have elevated IL-6 levels in serum and intraocular, although levels were not specifically correlated with a clinical diagnosis [24].

In one retrospective study, tocilizumab was found to be efficacious in treating uveitis patients with cystoid macular edema that was refractory to intraocular steroids or other immunosuppressive therapies [25]. These patients were found to have complete resolution after six months of therapy and were also found to have no recurrence of inflammation at follow-up, suggesting that it is able to maintain disease remission. In another recent case study, a patient with severe refractory posterior uveitis improved, with decreasing levels of IL-6 after treatment [26].

#### 3.2.4. Gevokizumab

IL-1*β* is an inflammatory cytokine produced in large amounts in Behcet's patients. Gevokizumab is a recombinant anti-IL-1*β* antibody, which modulates cytokine activity. It is a new therapy whose indications and efficacy are still being studied; a recent pilot study for patients with refractory Behcet's disease showed promising results, with only two infusions needed to render patients attack-free for several months [27]. Patients tolerated the infusions well, with no reported drug-related side effects. Treatment led to a rapid reduction in manifestations of intraocular inflammation, without the rebound attacks associated with discontinuation of corticosteroid use. This was thought to be in part due to accumulation of gevokizumab in ocular tissues, thus being able to sustain its therapeutic effect with an infrequent dosing interval.

### 3.3. Other

#### 3.3.1. Interferons

Interferons (IFN) are endogenous cytokines, released in response to external pathogens. IFN-*α* 2a, IFN-*α* 2b, IFN-*β* 1a, and IFN-*β* 1b are the classes most commonly used in therapy. Interferons are commonly used to treat conditions ranging from malignancy (cutaneous melanoma), infection (hepatitis C), and inflammatory (multiple sclerosis) [28, 29]. As far as ophthalmologic uses, IFN-*α* 2a has successfully treated Behcet's disease, and IFN-*β* 1a reduced uveitis recurrences in multiple sclerosis patients [30–33]. In Behcet's disease, interferon demonstrated significant benefit by decreases in aphthous ulceration and the number of lesions [34]. Several studies consistently reported that many patients had durable remissions of ocular inflammatory disease after discontinuation.

#### 3.3.2. Anakinra

Anakinra is an interleukin-1 receptor antagonist, which competitively inhibits IL-1 binding to its receptor. IL-1 has been found to have significance in systemic autoinflammatory diseases, where excessive IL-1 signaling will occur. It plays a key role in auto inflammatory diseases such as Muckle-Wells and neonatal onset multisystem inflammatory disease (NOMID), which are rare causes of uveitis in childhood [35]. It may in the future be used to treat refractory juvenile idiopathic and Behcet's disease, for which it is currently in phase III clinical trials [36]. 

## 4. Conclusion

Uveitis is a vision-threating group of diseases that encompasses a variety of etiologies, which are either infectious or noninfectious. Both groups are commonly treated with steroids. Uveitis resulting from infection, however, focuses on eradicating the source with antibiotics or antivirals. Those of noninfectious origin may need additional immunosuppressive agents. These antimetabolites, cytotoxic agents, biologics, and immunomodulators can be used either alone or together, to control inflammation of the vitreous. As with any medication, especially immunosuppressants, side effects must be balanced with therapeutic benefit—a determination still in process for many drugs and indications. The complexities in investigating these therapies result from the innate heterogeneity of uveitis. Even with its difficulties, research on expanding indications for existing therapies and the discovery of new systemic agents continues to progress.

## Figures and Tables

**Table 1:** Disease indications for immunosuppressive agents.

Strong Indications	Relative Indications
Behcet's disease with retinal involvement	Noninfectious uveitis
Vogt-Koyanagi-Harada syndrome	Retinal vasculitis with central vascular leakage
Sympathetic ophthalmia	Severe chronic iridocyclitis
Juvenile idiopathic arthritis-associated uveitis	Relapsing polychondritis with scleritis
Ocular manifestations of Wegener's granulomatosis	Ocular cicatricial pemphigoid
Rheumatoid necrotizing scleritis or peripheral ulcerative keratitis	Serpiginous choroiditis

**Table 2:** Immunosuppressive agents, organized into categories, and with information on mechanism of action, administration, side effects, and clinical management.

	Mechanism of action	Indications	Administration	Side effects	Management
Antimetabolites					
(1) Methotrexate	Folic acid analog; dihydrofolate reductase inhibitor, thus inhibiting synthesis of purines and therefore DNA, RNA, thymidylate, and proteins [7]. Reduces T-cell role in inflammation by inhibiting its activation and suppressing intercellular adhesion molecule expression [37]. With all administrations of methotrexate, it is critical to supplement folinic acid, to restore thymidylate and purine biosynthesis.	(i) Vitritis (ii) Vasculitides (iii) Anterior uveitis (iv) Orbital pseudo-tumor (v) Sarcoidosis	(i) Oral (ii) Subcutaneous (iii) IM (iv) IV Dose: 7.5–25 mg/week and May require 3–8 weeks for effects to take full effect. Course: two years after reduction of inflammation, to avoid recurrence [38].	(i) Common: fatigue, nausea, vomiting, and anorexia [39] (ii) Rare: hepatotoxicity, marrow suppression, and vasculitis (cutaneous) (iii) Teratogen Overall, long-term side effect profile is preferable compared to high-dose steroids.	Baseline: CBC, serum chemistry, BUN, Cr, LFT it, UA, pregnancy test. Follow-Up: CBC and LFT's every 4 weeks, with dose adjustment if LFT's double on two measurements. Stopped if LFT's stay elevated even after dose reduction [40].
(2) Azathioprine	Imidazolyl derivative; active metabolite is a purine synthesis inhibitor. Since lymphocytes have no method of nucleotide salvage, they are particularly affected [41].	(i) Serpiginous choroiditis (ii) Multifocal choroiditis (iii) Panuveitis (iv) Ocular cicatricial pemphigoid (v) Juvenile idiopathic arthritis [42–44]	Oral Dose: initially 2-3 mg/kg/day. Course: two years after reduction of inflammation, to avoid recurrence [45].	(i) GI upset (ii) Hepatotoxicity, bone marrow suppression, alopecia, and pancreatitis [46].	Baseline: CBC, LFT's, thiopurine methyltransferase enzyme activity (If low enzyme activity withhold treatment [46].) Follow-Up: CBC and LFT's every 4–6 weeks, with dose adjustment or temporary stop if abnormalities arise [47].
(3) Mycophenolate mofetil	Reversibly inhibits guanosine nucleotide synthesis, which particularly affects B- and T-cells [48]. it disrupts cellular adhesion to vascular endothelial cells, thus affecting lymphocytic chemotaxis [49].	(i) Chronic ocular inflammation [50] (ii) Scleritis, uveitis; used with cyclosporine and methotrexate [50].	(i) Oral (ii) IV Dose: initially 500 mg twice daily, thereafter increaseing to 1 g twice daily if well tolerated [45]. Course: two years following ocular quiescence [45].	(i) GI upset (nausea, vomiting, and diarrhea) (ii) Bone marrow suppression, hepatotoxicity [8]	Baseline: CBC, LFTs Follow-Up: CBC weekly for first month, twice monthly for next two months, and then monthly. LFT's monthly for duration of treatment [51].
(4) Leflunomide	Pyrimidine synthesis inhibitor, by inhibiting dihydroorotate dehydrogenase. In this manner, it suppresses B- and T-cell proliferation by interfering with cell cycle progression [52]. Nonlymphoid cells use a salvage pyrimidine pathway to synthesize ribonucleotides [52]. Leflunomide also has proven anti-inflammatory action, due to suppression of lymphocyte proliferation, tyrosine kinase, cyclooxygenase, and histamine release [53, 54].	Systemic rheumatology (severe rheumatoid and psoriatic arthritis). Ocular use in treating chronic inflammation associated with sarcoidosis is currently under investigation (see main text).	Oral Dose: loading dose100 mg and then 10–20 mg daily. A loading dose may result in initially increased adverse effects, but more rapid efficacy [55, 56]. To increase tolerability, patients may be given prednisolone rather than a loading dose [55]. Course: currently not certain.	(i) Serious hepatotoxicity (jaundice, hepatitis, and fatalities) (ii) Bone marrow suppression, interstitial lung disease, paresthesias, and headaches (iii) Teratogen [57] Due to its hepatotoxic effects, concurrent use with methotrexate is not recommended.	Baseline: CBC and LFTs. Follow-Up: both biweekly for the first six months, then bimonthly for the duration of treatment.

Alkylating agents					
(1) Cyclo-phosphamide	Cytotoxic properties are due to addition of an alkyl group to the guanine base of DNA and forming irreversible inter- and intrastrand DNA cross-links at guanine positions. This results in toxicity to rapidly-dividing cells (lymphocytes) and suppression of antibody production and delayed type hypersensitivity [58].	(i) Behcet's disease (ii) Polyarteritis nodosa (iii) Wegener's granulomatosis (iv) Mooren's ulcer [59–64]	IV Dose: starts at 1 g/m^2^ and adjusted on response and side effects [51]. At the beginning of treatment, given biweekly. Discontinued if hematuria occurs, with urology consult indicated if hematuria persists beyond three weeks [51]. Course: once ocular quiescence is achieved, space treatment intervals to every 3-4 weeks continued for 1 year.	(i) Bone marrow suppression (ii) Hemorrhagic cystitis (iii) Secondary cancers (bladder, AML) (iv) Testicular atrophy (v) Ovarian suppression (vi) Known teratogen	Baseline: CBC, LFTs, UA Follow-Up: CBC and urinalysis are initially repeated weekly then spaced out to monthly intervals when blood counts are stabilized.
(2) Chlorambucil	Cytotoxic properties from addition of an alkyl group and forming DNA crosslinks [65].	(i) Sympathetic ophthalmia (ii) Behcet's disease (iii) Serpiginous choroiditis [66, 67]	Oral Dose: two treatment algorithms. One starts at 0.1 mg/kg/day; maximum dosage 12 mg daily. The other uses short-term higher doses for 3–6 months [52]. Course: one year after ocular quiescence [47].	(i) Heme/Onc: myelosuppression, bone marrow aplasia, and secondary cancers (ii) Endocrine: male sterility, amenorrhea (iii) GI: hepatotoxicity (iv) CNS: seizures (v) Infectious: reactivation of latent herpes simplex virus [52, 68, 69].	Baseline: CBC w. differential, LFT's. Follow-Up: CBC initially repeated weekly, then spaced out to monthly intervals after stable dose. LFTs monthly.

T-cell inhibitors/calcineurin inhibitors					
(1) Cyclosporine	Suppresses T lymphocyte activity and thus the immune response. Binds lymphocytic protein cyclophilin, which inhibits calcineurin. Since calcineurin normally activates interleukin-2 transcription, there is decreased T lymphocyte function [70].	(i) Behcet's disease (ii) Sympathetic ophthalmia (iii) Sarcoidosis (iv) Birdshot retinochoroidopathy (v) VKH [71, 72]	Oral Dose: initially 2.5 mg/kg/day, increased in increments of 50 mg; maximum 5 mg/kg/day [47]. Course: two years after ocular quiescence [47].	(i) Hypertension, gingival hyperplasia, lymphoma nephrotoxicity (ii) Myalgia, tremor, or paresthesias	Baseline: LFT's, CBC w. differential, BUN, Cr, UA, blood pressure Follow-Up: blood pressure and electrolytes initially repeated biweekly spaced out to monthly after dose is stable. Other labs monthly [51].
(2) Tacrolimus	Macrolide antibiotic, whose mechanism is similar to that of cyclosporine; both inhibit calcineurin and suppress T-cell signaling and IL-2 transcription [73].	Used with systemic corticosteroids [73]. Often used when cyclosporine treatment fails [74, 75].	(i) Oral (ii) IV Dose: 0.10–0.15 mg/kg/day. The more serious adverse effects are seen at higher doses [76–78].	Hypertension, nephron-toxicity, electrolyte abnormalities, anorexia, neurologic (insomnia, confusion, depression, catatonia, tremors, and seizures), non-Hodgkin's lymphoma	Similar to cyclosporine.
(3) Rapamycin	Inhibits cellular response to IL-2 and inhibits activation of B and T lymphocytes. Rapamycin acts on “mammalian target of rapamycin” (mTOR), rather than on a calcineurin inhibitor, as cyclosporine and tacrolimus do.	Used with other immunosuppressive agents [79, 80].	Oral Dose: loading 6 mg; daily 2–6 mg/day [79].	Elevated LFT's, anemia, thrombocytopenia, hypercholesterolemia, nausea, abdominal pain, eczema, and increased risk of malignancy Markedly less nephrotoxic than other calcineurin inhibitors.	Similar to cyclosporine and tacrolimus

Biologic agents					
(1) Etanercept	Targets TNF-*α* and TNF-*β* receptor, preventing molecules from binding, thus inactivating TNF. Thus it suppresses neutrophil migration and cytokine synthesis.	Indeterminate; see paper	Subcutaneous Dose: 25 mg twice a week, for two years.	Infection, increased risk for latent TB and hepatitis B reactivation, CNS demyelination, pancytopenia, congestive heart failure, and lymphoma [81, 82].	Baseline: CBC, LFT's, TB skin test, hepatitis B serologic testing Follow-Up: monthly CBC and LFTs [52, 83].
(2) Infliximab	Binds to and inhibits TNF-*α* (bound or circulating) [84].	(i) Sarcoidosis (ii) Wegener's granulomatosis (iii) Juvenile inflammatory arthritis (iv) Behcet's disease [85–89]	Intravenous Dose: loading infusions weeks 0, 2, and 6; maintenance infusions every eight weeks [89]. For monotherapy, dose of 5 mg/kg; for concurrent noncorticosteroid treatment, dose of 3 mg/kg. Treatment for two years after ocular quiescence is achieved [40].	Infection (urinary tract, upper respiratory), GI (nausea, emesis), vasculitis, anemia, and thrombocytopenia [89–91].	Baseline: CBC, LFT's, TB skin test Follow-Up: monthly CBC and LFTs.
(3) Adalimumab	Binds to and inhibits TNF-*α* [92].	(i) Birdshot retinochoroidopathy (ii) VKH (iii) Behcet's disease (iv) Rheumatoid arthritis scleritis [12–16].	Subcutaneous Dose: 40 mg every two weeks [93]. Course: 2 years after ocular quiescence is achieved [40].	Injection site reactions, infections (urinary tract, upper respiratory), headache and confusion, CNS demyelination, hepatotoxicity, congestive heart failure, and lymphoma [94, 95].	Similar to infliximab.
(4) Daclizumab	Binds to CD25, a subunit of the IL-2 receptor on T lymphocytes [96].	(i) Birdshot retinochoroidopathy (ii) Posterior uveitis (iii) Juvenile inflamm arthritic uveitis [97–99].	Intravenous Dose: 1 mg/kg every two weeks; maximum daily dose of 200 mg [100]. Dose independent of concurrent immunomodulatory treatment. Course: two years after ocular quiescence is achieved [97].	Rash, lymphadenopathy, chest discomfort, and fever [101].	Baseline: CBC, LFTs Follow-Up: repeat baseline labs prior to each infusion.
(5) Rituximab	Binds to CD20, found on B lymphocytes. It thus suppresses B-cell differentiation, and decreased production of IgG and IgM [102].	(i) Wegener's granulomatosis [19] (ii) Retinal vasculitis [20] (iii) Ocular cicatricial pemphigoid [22]		(i) Death from infection (*Pneumocystis jiroveci*, progressive multifocal leukoencephalopathy) (ii) Toxic epidermal necrolysis (iii) Pulmonary toxicity [103, 104] (iv) Severe infusion reaction, cytokine release syndrome, and acute renal failure [22].	
(6) Tocilizumab	Blocks T/B-lymphocyte and monocyte IL-6 receptors, hindering its expression and proinflammatory effects. it increases Th1 cell specific regulatory binding protein of retinal photoreceptors, suggesting possible treatment of refractory uveitis associated with inflammatory or autoimmune processes [105].	(i) Rheumatoid and systemic juvenile idiopathic arthritis [23] (ii) Refractory uveitis [25]		(i) Common: infections, hypertension, headache, and transient increases in ALT [106] (ii) Rare: neutropenia, thrombocytopenia, GI (perforations or gastritis), infections (TB, fungal) [107]	
(7) Gevokizumab	Binds IL-1b and downregulates its activity.	Behcet's		None known currently	

Other					
(1) Interferons	Endogenous cytokines released in response to external pathogens.	Nonophthalmologic [28, 29]: (i) Melanoma (ii) Hepatitis C (iii) Multiple sclerosis Ophthalmologic [30–33]: (i) Behcet's disease (IFN-*α* 2a) (ii) Multiple sclerosis uveitis (IFN-*β* 1a)	Dose: IFN-*α* 2a given at 3–6 million international units, with frequency ranging from daily to three times weekly [108]. Course: maintain treatment after ocular inflammatory quiescence achieved for two years [7].	(i) Common: fever, chills, myalgias, alopecia, and depression [109]. (ii) Interferon retinopathy Unlike other immunosuppressants and biologic agents, IFNs rarely cause infectious complications and are also not carcinogenic.	Baseline: CBC, LFTs, and thyroid function tests Follow-Up: CBC and LFTs every four weeks; thyroid function tests every three months.
(2) Anakinra	IL-1 receptor antagonist; competitively inhibits binding of IL-1 to its receptor. IL-1 has been found to have significance in systemic autoinflammatory diseases, where excessive IL-1 signaling will occur [36].				

**Table 3:** Categories of vitritis drugs and what diseases they are indicated for.

Drug	Indications
Antimetabolites	
Methotrexate	Noninfectiouschronic uveitis, ocular inflammation, ocular sarcoidosis
Azathioprine	Chronic uveitis, Behcet's, choroidal neovascularization, ocular cicatricial pemphigoid, retinal vasculitis, serpiginous choroiditis
Mycophenolate mofetil	Chronic uveitis, noninfectious ocular inflammation, refractory uveitis, scleritis
Leflunomide	Sarcoidosis

Alkylating agents	
Cyclophosphamide	Refractory uveitis, nonnfectious ocular inflammation, ANCA-associated vasculitides
Chlorambucil	Serpiginous choroiditis, refractory uveitis, Behcet's

T-cell inhibitors/calcineurin inhibitors	
Cyclosporine	Serpiginous choroidopathy, Behcet's, scleritis, rheumatoid arthritis, nonnfectious uveitis
Tacrolimus	The above indications but usually in conjunction with systemic corticosteroids or adjunct immunosuppressants
Rapamycin	

Biologic agents	
Etanercept	Juvenile idiopathic arthritis, noninfectious uveitis, ocular inflammatory disease
Infliximab	Refractory uveitis, childhood uveitis, Behcet's
Adalimumab	Refractory uveitis, ankylosing spondylitis, juvenile idiopathic arthritis
Daclizumab	Juvenile idiopathic arthritis, recalcitrant ocular inflammation, birdshot chorioretinopathy
Rituximab	Primary Sjogren's syndrome, thyroid eye disease, Wegener's granulomatosis
Tocilizumab	Severe refractory posterior uveitis
Gevokizumab	Behcet's

Other	
Interferons	Behcet's, noninfectious uveitis
Anakinra	Behcet's, refractory juvenile idiopathic disease
